# Variant A of the Deformed Wings Virus Alters the Olfactory Sensitivity and the Expression of Odorant Binding Proteins on Antennas of *Apis mellifera*

**DOI:** 10.3390/insects12100895

**Published:** 2021-10-01

**Authors:** Diego Silva, Ricardo Ceballos, Nolberto Arismendi, Anne Dalmon, Marisol Vargas

**Affiliations:** 1 Laboratorios de Virología y Patologías en Abejas, Facultad de Agronomía, Universidad de Concepción, Av. Vicente Méndez 595, Chillán 3780000, Chile; diegosilva@udec.cl; 2 Laboratorio de Ecología Química, Instituto de Investigaciones Agropecuarias, INIA Quilamapu, Av. Vicente Méndez 515, Chillán 3780000, Chile; rceballos@inia.cl; 3 Centro de Investigación Austral Biotech, Facultad de Ciencias, Universidad Santo Tomás, Av. Picarte 1130–1160, Valdivia 5090000, Chile; narismendi@santotomas.cl; 4 Unité de Recherche Abeilles et Environnement, INRAE, F-84000 Avignon, France; anne.dalmon@inrae.fr

**Keywords:** honey bees, DWV-A, OBPs, antennae, olfactory responses

## Abstract

**Simple Summary:**

Honey bees, *Apis melllifera*, are the most commonly managed bee in the world for pollination services. However, worldwide continuous colony losses have been reported for almost a decade. One factor of these losses is associated to pathogens being the virus one of the most important problems in honey bee health. One of the known viruses that affect the honey bee population is deformed wing virus (DWV). DWV causes physical malformation and behavioral disturbances, but also, this virus can be found in the antenna affecting the anatomical integrity of infected areas, which could compromise normal antennal functioning associated to aroma perception. Thus, we evaluate olfactory sensitivity and the expression of antenna-specific odorant-binding proteins (OBP) genes in honey bees inoculated with variant A of the DWV. We performed olfactory sensitivity analysis using the essential oils *Eucalyptus globulus* and *Mentha piperita*, but also, and molecular analysis of gene expression of nine OBPs. We found that the high level of replication of DWV-A in the antennae decreased the olfactory sensitivity and led to a down-regulation of some OBPs in middle- and forager-age worker bees. Thus, DWV-A infection in adults of honey bees could compromise volatile compound recognition inside the hive and outside the hive.

**Abstract:**

Insects have a highly sensitive sense of smell, allowing them to perform complex behaviors, such as foraging and peer recognition. Their sense of smell is based on the recognition of ligands and is mainly coordinated by odorant-binding proteins (OBPs). In *Apis mellifera*, behavior can be affected by different pathogens, including deformed wing virus (DWV) and its variants. In particular, it has been shown that variant A of DWV (DWV-A) is capable of altering the ultra-cellular structure associated with olfactory activity. In this study was evaluated olfactory sensitivity and the expression of OBP genes in honey bees inoculated with DWV-A. Electroantennographic analyses (EAG) were carried out to determine the olfactory sensitivity to the essential oils *Eucalyptus globulus* and *Mentha piperita*. The expression of nine antenna-specific OBP genes and DWV-A load in inoculated bees was also quantified by qPCR. We observed an inverse relationship between viral load and olfactory sensitivity and the expression of some OBP proteins. Thus, high viral loads reduced olfactory sensitivity to essential oils and the gene expression of the OBP2, OBP5, OBP11, and OBP12 proteins on the antennas of middle- and forager-age bees. These results suggest that DWV-A could have negative effects on the processes of aroma perception by worker bees, affecting their performance in tasks carried out in and outside the colony.

## 1. Introduction

Pollination is one of the key processes in ecosystem services and agricultural production [1,2,3]. However, a significant decrease in the population of pollinating insects has been observed, particularly in *Apis mellifera* L. [4,5,6,7], attributed in part to pathogens that affect this species [4,8]. Among these pathogens, viruses are considered one of the most important problems in honey bee health [9,10]. In fact, compared to other pathogens, viruses can go unnoticed in honey bees because they often do not present clinical symptoms that allow their rapid detection [11]. One of the known viruses that affect the honey bee population is deformed wing virus (DWV). To date, three variants of DWV with epidemiological importance have been identified (A, B, and C); types A and C have been associated with winter colony losses in Europe [12,13,14,15].

DWV causes wing deformities, abdominal swelling, paralysis, and behavioral disturbances [15,16,17]. The DWV infection has been reported to alter molecular learning mechanisms, impairing the memorization process and altering the central and peripheral nervous systems [18]. Furthermore, DWV can replicate in the brain and alter the processing of sensory information [19]. Additionally, viral particles have been found in the optic and antennal lobes of the brain, which could compromise the vision and olfactory senses of honey bees [19,20]. More recently, it has been reported that variant-A (DWV-A) is capable of replicating in the epithelial cells of antennas, affecting the anatomical integrity of infected areas, which could compromise normal antennal functioning [21].

In fact, the olfactory system located mainly in the sensilla of the antennas plays a crucial role for insects, coordinating behaviors such as looking for and recognizing food sources, avoiding and identifying predators, and partner selection. Additionally, for social insects, such as bees, the olfactory system is essential in coordinating social events, beehive management, maintaining internal cohesion through the perception of pheromones, and defense-related behaviors [22,23]. The sensitivity, perception, and interpretation of aromas in the environment involve a great diversity of molecules that interact in the olfactory sensory cells; odor binding proteins (OBPs) are among these [24,25,26]. These small water-soluble molecules, located in the sensory dendrite, present selective binding to different compounds [25,27], such as hydrophobic odorants and pheromones, characteristic of environmental aromas [24,28]. The amount and diversity of OBPs vary among insect species, but in *A. mellifera*, a total of 21 OBPs have been identified, with only nine of the specific expression in the antennas [24]. Thus, the presence of DWV in the antenna could intervene in aroma recognition processes, possibly due to alterations in the expression of OBPs. For these reasons, the aim of this study was to evaluate the olfactory perception and gene expression of OBPs in antennas of worker bees that were inoculated with variant A of the deformed wing virus (DWV-A), with non-inoculated worker bees as a control. This study furthers our understanding of the magnitude of harm caused by the DWV-A infection in the antennas of honey bees and how this infection affects olfactory perception and could possibly alter bee behavior at the individual and colony level.

## 2. Materials and Methods

### 2.1. Inoculum Preparation

An isolate of deformed wing virus variant-A (DWV-A) was obtained from infected colonies, according to Gusachenko et al. [29], and was subsequently used. A group of 20 bees was homogenized in a Stomacher bag with phosphate-buffered saline (1X PBS) for 90 s at high speed in a Stomacher 80 Lab Blender (Seward, London, UK). The samples were centrifuged twice at 1500× *g* for 10 min, followed by 10,000× *g* for 10 min, both at 4 °C. The supernatant was purified by filtration with a 0.22 µM filter (PES, Merck Millipore, Darmstadt, Germany) and treated with RNase to destroy all unencapsidated RNA, according to Skubnik et al. [30]. Later, 200 µL of the supernatant was used for RNA extraction, cDNA synthesis, and the subsequent viral load quantification of the inoculum, according to the methodology detailed in the section “RNA extraction, cDNA synthesis, and real-time PCR quantification (qPCR)”. The rest of the extracted inoculum was stored at −80 °C until later use.

### 2.2. Bee Inoculation

Adult *A. mellifera* for the assay were supplied by the experimental apiary located in the Experimental Center “El Nogal” (36°35′58.25″ S–72°04′51.77″ W), Universidad de Concepción, Chillán, Chile. Before starting the assay, the health status of the apiary was determined by identifying the viral level in each colony (*n =* 54 beehives). Thus, the pathogen level was determined by molecular techniques, according to Vargas et al. [31]. Brood combs with capped worker bees were then removed from colonies with low DWV (1.0 *×* 10^2^ copy number per bee), considering that no colonies were detected DWV-free. The brood combs were maintained under controlled conditions in a rearing room (30 °C ± 1; 60% ± 3 RH). Afterwards, newly emerged worker bees (24 h old) were carefully collected from the brood combs and randomly confined in plastic cages (base = 8 cm diameter, mouth = 10 cm diameter, and height = 15 cm). Two treatments were then established. One group of bees were inoculated (I-DWV treatment) orally with 5 µL of a viral suspension (1.0 *×* 10^9^ copy number per bee) in a 50% sucrose solution, according to the methodology reported by Porrini et al. [32]. Another group of bees were not inoculated with the viral suspension (N-DWV treatment), as a control. Those bees that did not consume the total amount of viral inoculum were discarded from the assay. Then, 60 worker bees were maintained in plastic cages (3 replicates per treatment) containing 3 g of pollen substitute and supplied with 60% sucrose syrup ad libitum, according to Arismendi et al. [33]. Five bees were removed from each cage of each treatment every 3 days, up to a maximum of 21 days post-inoculation (23-day-old bees).

### 2.3. Stimulus Preparation

As a stimulus, the volatile fraction of the essential oils of *Mentha piperita* and *Eucalyptus globulus* leaves, both described as forage species of *A. mellifera,* were used [34,35,36,37]. In the summer of 2019, we collected leaves from ornamental specimens of both species in Chillán, Ñuble Region, and Chile and obtained 270 and 365 g from *M. piperita* and *E. globulus*, respectively. The leaves were air-dried at room temperature for 48 h and then boiled for 8 h to obtain the essential oil by hydro-distillation using a Clevenger apparatus [38]. After this extraction, we added sodium sulfate anhydrous (Na_2_SO_4_) to remove water from the samples, after which they were stored at 8 °C in complete darkness. We prepared four diluted concentrations in hexane (≥99% GC, Sigma-Aldrich, Munich, Germany) at 0.01, 0.1, 1.0, and 10.0 mg/mL as stimuli for the electrophysiological test.

### 2.4. Antennal Response

The olfactory sensitivity of adults of *A. mellifera* was evaluated by electroantennography (EAG). The antennas, stimulus receptor organs, were used for the experiment once they were removed from the sampled individuals. One antenna was immediately stored at −80 °C, and the other was used in the EAG record. After testing, this antenna was returned and stored at −80 °C for further molecular analyses. The antennas were extirpated on the base of the scape with a scalpel, and the first segment of the distal end was cut with dissecting scissors to improve the connection. The excised antennas were mounted between glass electrodes filled with a conductive saline solution of KCl (0.1 M) and 0.1% polyvinylpyrrolidone [39]. A volume of 10 µL of stimuli, at different concentrations, was deposited on filter paper (Whatman N°1, Whatman^®^, Sigma-Aldrich, Darmstadt, Germany) and was then delivered to the antenna at 40 mL/s; thus, the duration of each stimulation was 0.2 s using a stimulus controller (CS-55, Syntech, Hilversum, The Netherlands), with a period of at least 30 s between each stimulation to permit the recovery of the antennas. Each antenna was stimulated 3 times for each concentration of essential oil; additionally, each antenna was stimulated with a target (air) and a control (Hexane ≥99% GC, Sigma-Aldrich, Munich, Germany). The data were recorded using the EAG 2014 Software (Syntech, Hilversum, The Netherlands) for the acquisition and analysis of the depolarizations obtained during the electroantennography.

### 2.5. RNA Extraction and cDNA Synthesis

For the RNA extraction and subsequent quantification of viral load (DWV) and OBP gene expression, a pool of 10 antennas from 5 bees (including antennas used in the electroantennography assay) were used. The RNA extraction was carried out according to Kim et al. [21] and Mondet et al. [20]. In brief, the antennas were cooled with liquid nitrogen and then, were ground by adding 500 µL of Trizol^TM^ (Invitrogen, Thermo Fisher Scientific, Waltham, MA, USA) and 5 µL of Carrier RNA, according to the manufacturer’s recommendations (Invitrogen, Thermo Fisher Scientific, Waltham, MA, USA). The homogenate was incubated in the cold for 5 min, then it was transferred to a 1.7 mL tube, and 100 µL of chloroform was added and incubated at 4 °C for 3 min. It was then centrifuged for 15 min at 10,300× *g* at 4 °C, and finally, the supernatant was collected for the RNA extraction. The RNA extraction was carried out following the instructions provided by E.Z.N.A. Total RNA kit I (Omega Bio-Tek, Norcross, GA, USA). RNA quality and yield were determined using a spectrometer (Infinite 200 PRO NanoQuant, Tecan Group, Männedorf, Switzerland), which was stored at −80 °C. The extracted RNA was subsequently used for first-strand cDNA synthesis, by using the enzyme reverse transcriptase M-MLV (Invitrogen, Life Technologies, Carlsbad, CA, USA), according to the manufacturer’s instructions. The cDNA samples were stored at −20 °C for later use.

### 2.6. Real-Time PCR Quantification (qPCR)

To quantify the viral load and expression of OBP genes, specific primers were used (Table 1). The PCR reaction was carried out using 1× of KAPA SYBR FAST Universal 2× qPCR Master Mix (Kapa Biosystems, Wilmington, MA, USA), following the supplier’s instructions. Samples were brought to a reaction volume of 15 µL, including 20 ng of cDNA, 530 nM of each primer, and sterile filtered molecular-grade water to reach 15 µL. The thermal reaction conditions used were 96 °C for 10 min, followed by 40 cycles at 95 °C for 15 s, 60 °C for 15 s, and 72 °C for 30 s. Real-time PCR assays were performed on a Stratagene Mx3000P thermal cycler (Agilent Technologies, Santa Clara, CA, USA), and the data were analyzed using MxPro software (Stratagene, Agilent Technologies, Santa Clara, CA, USA). The relative expression of each OBP gene was calculated after normalization with an endogen gene (β-actin), as described by Pfaffl [40]. Similarly, to determine the viral load in the antennas, absolute quantification of DWV-A was performed. In brief, a standard curve using purified PCR product (Wizard^®^VR SV gel and PCR clean-up system, Promega, Madison, WI, USA), belonging to the viral target sequence, was used [41]. The purified amplicon was then quantified by spectrophotometry (EpochTM Microplate Spectrophotometer, BioTek, Winooski, VT, USA) to calculate the copy number, according to Wu et al. [42]. Thus, linear standard curves (95–100% efficiency) were then generated using a serial dilution (1.0 *×* 10^1^ to 1.0 *×* 10^9^) of viral copy numbers of purified cDNA. Afterwards, the Ct values were plotted against copy number values (log_10_). Thus, the sample copy numbers were estimated using the Ct values and comparisons with the linear equation of the standard curve and normalizing values of the housekeeping β -actin gene [43]. Data were then expressed as the copy number of DWV per bee by considering the dilutions that were performed in the cDNA synthesis and qPCR reaction.

### 2.7. Data Analysis

To meet the assumptions for normality and homogeneity of variances, the EAG values were transformed to square roots (√x). A general linear model (GLM) was then used to design a split-split-plot ANOVA with data of the EAG response. Thus, honey bee age (2, 5, 8, 11, 14, 17, 20, and 23 days old) was considered as the main factor (and levels), and the (1) essential oil concentrations (0.01, 0.1, 1.0, and 10.0 mg/mL) and (2) viral status (bees inoculated and non-inoculated with DWV-A) were considered as split factors in function to the EAG response (response variable) to stimuli elicited by the volatile fractions of essential oils from *E. globulus* and *M. piperita*. The Bonferroni test (*p* < 0.05) was then run to separate means between treatments.

Statistical differences in the viral loads in honey bees according to (1) viral status (non-inoculated bees collected from frames that were detected with a basal low level of DWV-A (N-DWV) and those that were additionally inoculated with DWV-A (I-DWV)) and (2) bee ages (honey bees 2 to 23 days old) were estimated by Factorial ANOVA. Tukey’s HSD test (*p* < 0.05) was then used to separate means between treatments.

Considering that OBPs expression values were correlated in bees that were inoculated and non-inoculated with DWV-A, a multivariable analysis (MANOVA) was carried out. Thus, honey bee age and viral status were considered independent variables in function to OBPs relative expression (response variable). After this, means were separated between treatments with a Bonferroni test (*p* < 0.05). In cases that detected statistical differences, the results were graphically represented on a heat-map illustrating the intensity of the OBPs gene expression in the antennae of worker honey bees that were inoculated with DWV-A in contrast with non-inoculated bees at different ages.

Additionally, Spearman’s correlations were performed to estimate relationships among the viral load, the relative expression of OBP genes, and EAG values in DWV-A inoculated bees. For the Spearman correlation, the data from worker bees that were 8 days old or more were considered because, from this period on, the viral load was significantly higher in inoculated bees compared to those that were not inoculated. All analyses were carried out with STATISTICA 7.0 software (StatSoft, Tulsa, OK, USA).

## 3. Results

### 3.1. Electroantennography Responses and Viral Load in Antennas

**Figure 1:**
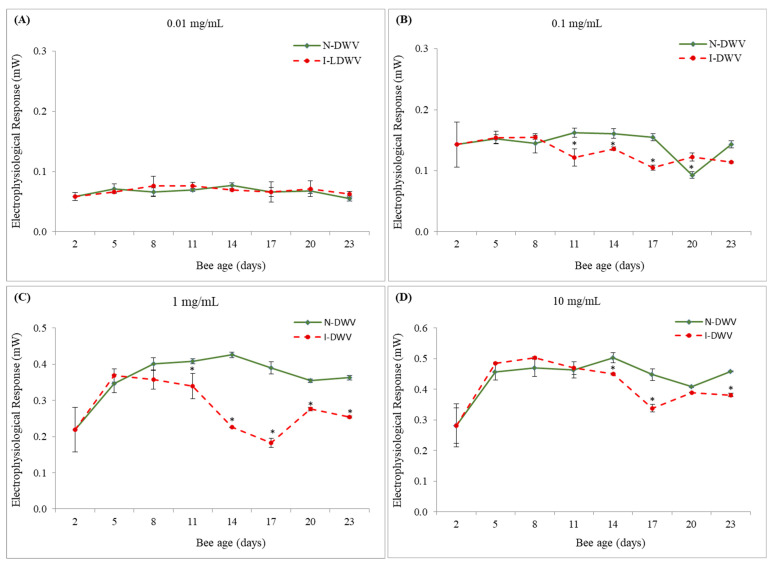
Electrophysiological response of inoculated and non-inoculated worker bees exposed to essential oil of *Eucalyptus globulus*. Uppercase letter in each subfigure represent the essential oil at concentration of (**A**) 0.01, (**B**) 0.1, (**C**) 1.0 and (**D**) 10.0 mg/mL. Asterisks indicate significant differences for the same sampling time in bees that were inoculated (I-DWV) and non-inoculated (N-DWV) with DWV-A, according to the Bonferroni test (*p* < 0.05). The bar in each age indicates standard error.

When inoculated worker bees (I-DWV) and non-inoculated bees (N-DWV) of different ages post-emergence (2, 5, 8, 11, 14, 17, 20, and 23 days old) were exposed to volatile stimuli of essential oils of *E. globulus* and *M. piperita* at different concentrations, a significant interaction between the viral status of worker bees and the essential oil concentration was found (*E. globulus* F = 5.73; df = 3, 64; *p* = 0.002 and *M. piperita* F = 4.25; df = 3, 64; *p* = 0.008). On the other hand, there was no significant interaction between bee age, viral status, and the essential oil concentration (*E. globulus* F = 0.62; df = 21, 64; *p* = 0.892 and *M. piperita* F = 0.77; df = 21, 64; *p* = 0.743). Bees that were inoculated with DWV-A (I-DWV treatment) responded less to stimuli of essential oils of *E. globulus* at concentrations of 0.1, 1.0, and 10.0 mg/mL, especially in bees that were 11 to 17 days old (Figure 1B–D), than bees that were not inoculated with DWV-A (N-DWV treatment). In fact, inoculated bees (I-DWV) from 14 to 17 days old, responded between 47% and 53% less compared to non-inoculated bees when exposed to essential oils at concentrations of 1.0 mg/mL (Figure 1C). Furthermore, this negative response in olfactory sensibility was also observed in older bees (20 and 23 days old), especially at concentrations of 1.0 and 10.0 mg/mL (Figure 1C,D). On the other hand, no significant differences were observed in the electrophysiological responses of antennas exposed to the essential oil of *E. globulus* at a concentration of 0.01 mg/mL (Figure 1A).

**Figure 2:**
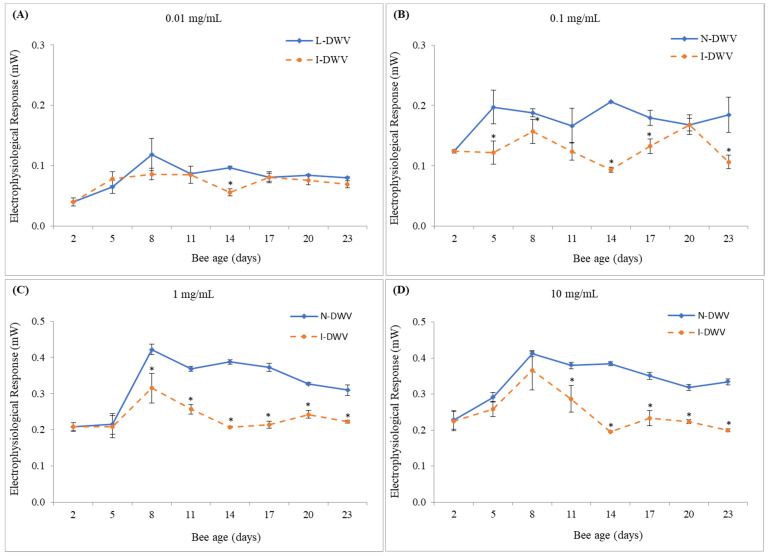
Electrophysiological response of inoculated and non-inoculated worker bees exposed to essential oil of *Mentha piperita.* Uppercase letter in each subfigure represent the essential oil at concentration of (**A**) 0.01, (**B**) 0.1, (**C**) 1.0 and (**D**) 10.0 mg/mL. Asterisks indicate significant differences for the same sampling time in bees that were inoculated (I-DWV) and non-inoculated (N-DWV) with DWV-A, according to the Bonferroni test (*p* < 0.05). The bar in each age indicates standard error.

Similarly, antennas stimulated with the volatile fraction of the essential oil from *M. piperita* also showed significant differences in bees that were inoculated with DWV-A (I-DWV) compared to those that were not inoculated (N-DWV) (Figure 2). However, these differences were mostly observed at 0.1, 1.0, and 10.0 mg/mL of essential oil from *M. piperita*, where worker bees that were inoculated with DWV-A showed a lower response to volatile compounds than non-inoculated bees, especially bees from 8–11 to 23 days old (Figure 2B–D). In some cases, these reduced responses to essential oils of *M. piperita* were, on average, 50% less than bees that were not inoculated with DWV-A (N-DWV treatment), especially in 14 day-old bees (Figure 2B–D). A significant difference in the electrophysiological responses of antennas exposed to the essential oil of *M. piperita* at a concentration of 0.01 mg/mL was also observed (Figure 2A). However, this difference was punctual and only observed in DWV-A inoculated 14 day-old bees, which showed a lower electroanemographic response to the volatile compounds of the essential oil of *M. piperita* (Figure 2A).

**Figure 3:**
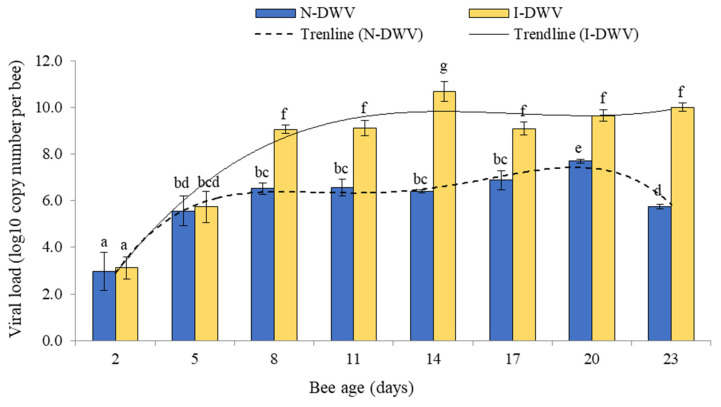
DWV-A load measured in antennae of worker honey bees of different ages that were inoculated (I-DWV) and non-inoculated (N-DWV). Means (±SE) with different letters indicate significant difference according to the Tukey HSD test (*p* < 0.05). Values of DWV-A load in 2-day old bees indicate the basal level of viral infection before the inoculation.

The viral load detected in antennas varied significantly between bees that were inoculated and non-inoculated; these changes were influenced by bee ages (Factorial ANOVA F = 7.78; df = 7, 32; *p* < 0.001). These differences were observed in 8 to 23-day-old bees, when bees inoculated with DWV-A (I-DWV treatment) showed a higher viral load (1.0 *×* 10^9^ to 1.0 *×* 10^11^ copy number per bee) compared to bees that were not inoculated (N-DWV treatment) (1.0 *×* 10^6^ to 1.0 *×* 10^7^ copy number per bee) (Figure 3).

### 3.2. Expression of OBP Genes

**Figure 4:**
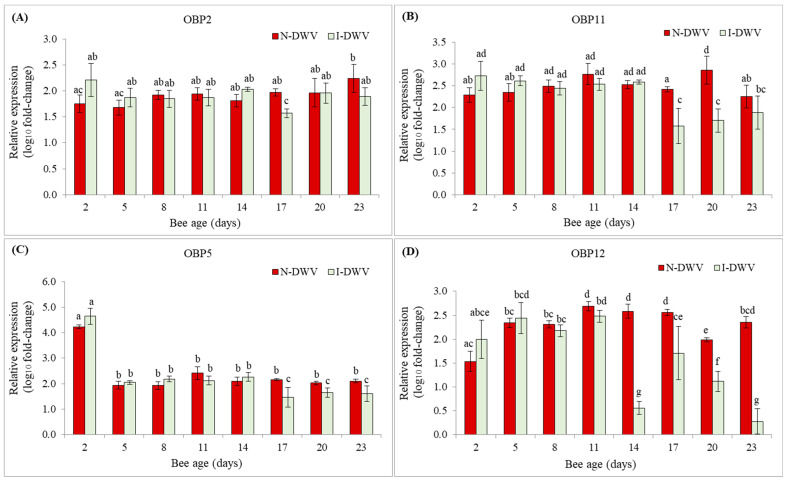
Gene expression of OBPs in worker honey bees of different ages and that were inoculated (I-DWV) and non-inoculated (N-DWV) with DWV-A. Means (±SE) with different letters indicate significant differences according to the Bonferroni test (*p* < 0.05) in (**A**) OBP2, (**B**) OBP11, (**C**) OBP5 and (**D**) OBP12.

**Figure 5:**
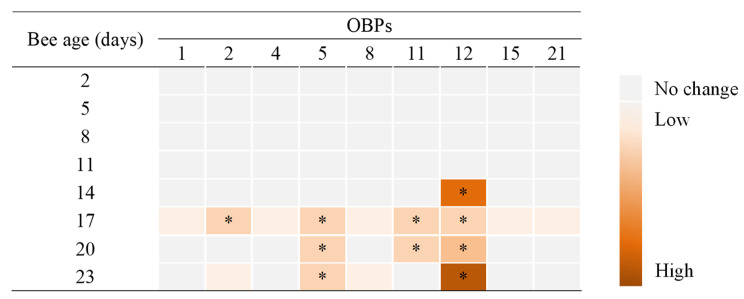
Heat-map illustrating the intensity of down-regulation of odorant binding proteins (OBPs) in the antennae of worker honey bees of different ages that were inoculated with DWV-A. Asterisks indicate significant differences (Bonferroni test, *p* < 0.05). Gene expression of OBPs in antennae of inoculated bees with DWV-A compared to non-inoculated bees.

Regarding the relative expression of the nine antenna-specific OBP genes of worker bees of *A. mellifera*, we observed significant differences in four of them; OBP2, OBP 5, OBP11, and OBP12 (Figure 4). However, changes in these gene expressions were an effect of the interaction between bee age and viral status (MANOVA Wilks λ = 0.01; F = 2.53; df = 70, 141; *p* < 0.001). In bees that were inoculated with DWV-A, OBP5, and OPB11, gene expression was significantly down-regulated compared with non-inoculated 17-day-old bees, a trend that continued until the end of the experiment (Figure 4B,C and Figure 5). Similarly, the OBP12 gene expression was down-regulated in inoculated bees that were 14 to 23 days old (Figure 4D and Figure 5). The remaining OBPs that were tested were not significantly altered by the viral level in the antenna (Figure S1, Supplementary Materials).

Correlations among the viral load, OBPs gene relative expression (OBP2, OBP5, OBP11, and OBP12), and EAG values (*M. piperita* and *E. globulus* at 0.1, 1.0, and 10.0 mg/mL) in DWV-A inoculated bees were identified using the Spearman correlation test (Table 2). Significant negative correlations were detected among the viral load and OBP12 expression (r_s_ = −0.69, *p* = 0.001). Similarly, the increase of the viral load was negatively correlated with the olfactory response of bees inoculated with DWV-A to *M. piperita* volatile compounds at different concentrations (0.1 mg/mL r_s_ = −0.76, *p* < 0.001; 1.0 mg/mL r_s_ = −0.70, *p* = 0.001; 10.0 mg/mL r_s_ = −0.83, *p* < 0.001). On the other hand, there were no detected significant correlations among viral load, the expression of OBP5 and OBP11, or the response of bees that were inoculated with DWV-A to *E. globulus* volatiles (Table 2).

## 4. Discussion

To the best of our knowledge, this is the first evidence demonstrating that the olfactory sensitivity, evaluated by EAG, is negatively altered in in the antenna of bees inoculated with DWV-A. However, this alteration in the olfactory sensibility was dependent on the DWV-A titer in the antenna and the concentration of the stimuli component. Thus, the olfactory responses could be associated with the volatile compounds present in the essential oils from the plant species used in the study. Previous reports have shown that the presence of compounds, such as α-pinene, myrcene, linalool, and α-terpineol, in both essential oils [45,46,47,48] could be responsible for the EAG response since these compounds have been reported to stimulate the olfactory response in *A. mellifera* when individually exposed [49,50,51].

The antennae of bees were more sensitive to volatile compounds of essential oils of *M. piperita* than to those of *E. globulus*. In fact, the increase in the viral load of DWV-A in the antennae was negatively and significantly correlated with the electrophysiological response (EAG) of honey bees exposed to volatile essential oils of *M. piperita* (Table 2). This olfactory response was visible in 8-day-old bees and older, when the viral concentrations were also significantly higher (1.2 *×* 10^9^ copy number per bee) in bees that were inoculated with DWV-A (Figure 1 and Figure 2). We observed that, in general, the olfactory sensitivity of DWV-A inoculated and non-inoculated bees to essential oil concentrations of 0.1 and 1.0 mg/mL showed clearly differential responses. At high concentrations of essential oils, the olfactory response may be more irregular and could depend on the source from which it was obtained. For instance, essential oils of *E. globolus* at 10.0 mg/mL caused an irregular response in bees that were inoculated with DWV-A, although some cases were significant compared to non-inoculated bees. This irregular response could be associated with a saturation of the environment by the volatiles present in the essential oils, generating nonspecific stimulation [52]. It has been proposed that, when faced with a high concentration of stimulus, a nonspecific stimulation can be generated; on the other hand, exposure to low concentrations of the stimulus may not generate a complete stimulation of the antenna [52]. We observed that very low concentrations of the stimulus did not induce olfactory responses, although, exceptionally, 14-day-old bees inoculated with DWV-A and exposed to 0.01 mg/mL of *M. piperita* essential oil showed a significant decrease in the EAG response; bee age also coincided with a higher DWV-A load (1.0 *×* 10^11^ copy number per bee) detected in the antennas (Figure 2A and Figure 3). In fact, we observed that with an increase in the DWV-A load in the antennae, even at high concentrations of stimulus, the olfactory response decreased substantially (Figure 2D and Figure 3).

There exists the possibility that this negative effect on the olfactory response could be associated with damage to specialized structures in the antenna. In fact, Kim et al. [21] observed an alteration in the cellular ultrastructure of the antennas in bees with classic symptoms (e.g., deformed wings) and high viral loads (1.0 *×* 10^14^ copy number per bee). In contrast, non-symptomatic bees showed no damage to the cellular ultrastructure of the antennas, although the viral load found in this sensorial organ could also be considered high at 1.0 *×* 10^11^ copy number per bee. We did not observe common symptoms of the DWV in the bees used in the experiment; even so, we quantified considerably high viral particle levels in DWV-A inoculated bees (Figure 3), which caused significant negative sensorial effects; effects that could be even greater in symptomatic bees, as suggested by Kim et al. [21]. Thus, high levels of DWV in the antenna may affect the performance of this organ in terms of the perception of environmental ligands. Mondet et al. [20] showed that DWV infection in antennas compromised the development of Varroa Sensitive Hygiene (VSH) behavior, possibly due to the deterioration of the olfactory signal and damage in cognitive functions, which suggests that DWV may seriously compromise the processes involved in the perception of volatile compounds.

The negative effect on the olfactory response could also be associated with the gene expression of olfactory sensory proteins, such as OBPs. We found that the DWV-A infection significantly affected the gene expression of some OBPs located in the antenna. High levels (>1.0 *×* 10^9^ copy number per bee) of DWV-A in the antennas of middle-aged bees (8–23 days old) generated a decrease in the expression of OBP2, OBP5, OBP11, and OBP12, which could then cause the decrease in olfactory sensitivity. Previous studies have reported on the fundamental role of these proteins in the transport and solubility of environmental ligands that enter the lymph and, through the action of OBPs, are transported to chemosensitive neurons, especially due to their high concentration in the lymphatic zone [26,53,54,55]. This guarantees the detection and discrimination of chemical signals, as well as the sensitivity of the olfactory system [54,56,57]. In particular, the affinity and selectivity of the OBP2 protein in plant odors has been demonstrated, showing its physiological role in the recognition of forage species, especially in those that present compounds such as 1–8 cineole in their aroma [58]. Additionally, Zhao et al. [59] reported the crucial role of the OBP5 protein in the larval care processes within the hive. This protein has an affinity for volatile compounds released by larvae when they are affected by different pathogens, suggesting that the recognition of these volatile compounds by nurse bees could aid in recognizing diseased larvae and caring for health larvae, being key in the sanitary maintenance of the brood within the hive. Previously, Zhao et al. [60] also showed that OBP11 protein is produced in greater quantity in worker bees that perform tasks within the hive, reaching their peak gene expression in bees 10 to 15 days old, suggesting that this OBP is related to the olfactory detection of volatile compounds in the brood. However, Nie et al. [61] found that OBP2, OBP5, and OBP11 are expressed continuously from mid- to high levels in newly emerged worker bees, nurse bees, and forager bees. It is well known that 4- to 12-day-old worker bees feed larvae, while 12- to 20-day-old workers have many tasks, ranging from nest building and maintenance to nectar receiving and processing, and other labors associated with colony defense (e.g., guardian bees) [62]. Many of these tasks are mediated by pheromones and other secreted volatile compounds by queen and worker bees [63]. We observed that OBP5 and OBP11 were down-regulated significantly in bees 17 days old and older (Figure 4) when they were inoculated with DWV-A. This suggests that high levels of DWV-A infection in the antennae may affect many worker bees’ tasks, not only those described by Zhao et al. [60], but also other tasks associated with middle- and older-age bees [62], since these OBPs were expressed continuously in bees more than 15 days of age (Figure 4). On the other hand, there is little information on the specific olfactory role of the OBP12 protein. However, Nie et al. [61] also found that OBP12 is expressed continuously in antennae of newly emerged worker, nurse, and forage bees, which suggests that this protein could play an important role in the worker bees’ performance in the perception of aromas in and outside the hive. Furthermore, it was the only OBP in which gene expression was negatively and significantly correlated with the increasing DWV-A load (Table 2), which suggests that there is a strong relationship between DWV-A infection and the performance of aroma perception mediated by OBP12 in the antennae. In fact, the OBP12 was the most sensitive to DWV-A inoculation; its expression was down-regulated strongly in 14-day-old bees, until the end of the experiment (23-day-old bees) (Figure 5). This evidence suggests that this OBP may play key roles in middle-aged bees associated with maintenance tasks within a colony, but also with foraging bee activities outside the hive, such as locating and selecting food sources. However, new studies are required to support this hypothesis since there is no evidence of the specific functions of OBP12 in worker bees of different ages. Furthermore, we could be underestimating the effect of DWV-A on the gene expressions of OBP2, OBP5, OBP11, and OBP12, considering that the down-regulation of these OBPs was significantly observed, in most cases, in bees 17 days old and older. But these effects could also be related to the methodological procedure, in the sense that, in this study, newly emerged adult bees were inoculated orally, which reflects only one possible route of viral infection (e.g., trophallaxis). If the viral inoculation occurred in the pupa stage, simulating a transmission route via *Varroa destructor,* the results could be different. Therefore, viral infection of bees in immature stages could lead to a low expression of these OBPs (OBP2, OBP5, OBP11, and OBP12), much lower than the levels detected here; the expression of other OBPs that showed no significant changes in this study could also be affected in this case (Figure S1, Supplementary Materials). Hence, further studies are required to test this hypothesis, including inoculation with DWV-A in immature bee stages in order to more precisely measure OBPs expression and other odorant-associated genes present in the antennae of newly emerged, middle-aged, and forager bees.

It has been hypothesized that the presence of DWV-A may compromise the maturation process of worker bees from nurse to forager bees, particularly due to the change that this virus generates in the brain transcriptome. A recent study reported that nurse bees infected with DWV-A showed a brain transcriptome similar to forager bees and this change in behavioral maturation could be generating younger bees with foraging behavior [64]. However, this early foraging activity in middle-age bees could be precarious and deficient [65]. Thus, an DWV-A infection could impair worker bees’ olfactory sensitivity to aromas, as shown in this study, and a neurogenomic spoilage [64] could result in learning deficits in worker bees [18] that could lead to the loss of honey bees in the field (e.g., forage bees) and affect the efficiency of bees’ labors within the hive, producing a gradual decline in the colony, as has been reported with this DWV variant [66].

An interesting result in our study was that the difference in DWV-A load in the antennae between inoculated and non-inoculated bees was only observed in bees that were 8 days old or more (Figure 3). We expected that 3 days after (5-day-old bees) the DWV-A inoculation, the viral load in the antennae would be higher in inoculated bees than non-inoculated bees; however, no significant difference was observed at that time (Figure 3). Notwithstanding, when we analyzed and quantified the viral load in the rest of the body tissues (including head, thorax, and abdomen) of worker honey bees from which the antennas were removed, we found that the DWV-A load was significantly higher in 5 day-old inoculated bees (1.0 *×* 10^8^ copy number per bee) than non-inoculated bees (1.0 *×* 10^6^ copy number per bee) (Figure S2, Supplementary Materials). This shows that the DWV-A establishment and replication occurred only a few days after the inoculation. This result suggests that the DWV-A distribution process towards the antenna and its replication there occurs a little later than in other tissues or organs that have immediate contact (e.g., gut tissues) with the virus when it is inoculated orally. It was also interesting to observe that the DWV load in non-inoculated bees, which reflects the viral infection of the colony, was, on average, over time (1.0 *×* 10^6^ copy number per bee) in antennae and the rest of the analyzed bee body (Figure 3, Figure S2, Supplementary Materials); values that are considered low are in [64]. Although no DWV-free colonies were found in our experimental apiary, we used frames from colonies with very low DWV-A, without varroa or other pathogens for the experiment in this study, a strategy that has also been used in other related studies [67,68,69]. In fact, DWV is widespread in low levels in almost all honey bees, including *Varroa*-free colonies [70]. Honey bees can live with these basal low levels of DWV, which are apparently not harmful for them [71], although other stressful factors (e.g., ectoparasite *V. destructor*, gut parasite *Nosema ceranae*, poor nutrition, etc.) can eventually induce an increase in these levels and cause damage to the bees [69,72,73,74].

Our study provides novel information that aids in understanding the magnitude of harm generated by the DWV-A infection in middle- and forager-age worker bees. The presence of this virus in *A. mellifera* antennas could generate deterioration in crucial tasks inside the hive, such as larval care, cleaning, and defense activities. This damage could also extend outside the hive, affecting the forager bees and their ability to recognize food sources. However, further studies are required to understand and quantify the damage caused by DWV-A on behavioral responses of honey bees at the field level, considering the other DWV variants in the study model.

## 5. Conclusions

In summary, the high level of replication of DWV-A in the antennae decreased the olfactory sensitivity to the evaluated volatile compounds in middle- and forager-age worker bees. DWV-A also led to a down-regulation of some OBPs that are specifically expressed in antennas. Thus, DWV-A infection could compromise volatile compound recognition inside the hive and outside the hive in bee foraging behaviors, affecting bee survival at the individual and colony levels.

## Figures and Tables

**Table 1:** Primers used in the qPCR amplification for the odorant binding proteins (OBPs), the β- Actin gene, and specific primer to variant A of deformed wing virus (DWV-A).

Primer		Sequence (5′–3′)	References
AmelOBP1	F	ACCTGGTAAACGAACCGTCCA	[23]
	R	TCAACACAGCCTGTTCTCGA	
AmelOBP2	F	TCTGACCGTTGTACGTGGCA	[23]
	R	TGGCATTCTCGATGCACTCA	
AmelOBP4	F	TGCGCTGGTTCACGCAGACA	[23]
	R	ATGCATTCGTCTTCGTCTGCA	
AmelOBP5	F	ATGCGGAAATCGTGCTTGCA	[23]
	R	TGCCATTACTCACGGGAAGA	
AmelOBP8	F	GTTGCGTGGCAATTTGGCAAATG	[23]
	R	TACGTTGTTTCGCCCTTCCAGT	
AmelOBP11	F	TGAGGATGTCGAAGCTACGGAA	[23]
	R	CACGGAGCAATAAACGCTATGG	
AmelOBP12	F	TGCGTGGATCGATCAAACATGA	[23]
	R	ACGTTAACGCGATCTTATGGA	
AmelOBP15	F	TTGCATGGCAAAAACTGGCA	[23]
	R	TCTCTGGATACGTGTTCGTTGA	
β-Actin	F	ATGCCAACACTGTCCTTTCTGG	[43]
	R	GACCCACCAATCCATACGGA	
DWV-A	F	TATCTTCATTAAAGCCACCTGGAA	[44]
	R	TTTCCTCATTAACTGTGTCGTTGAT	

**Table 2:** Spearman correlation analysis between the viral load, OBPs gene relative expression, and EAG values in DWV-A inoculated worker honey bees.

First Variable	Second Variable	r_s_	t-Value	*p*-Value
DWV-A load	Relative expression			
	OBP2	0.45	2.03	0.059
	OBP5	0.25	1.04	0.316
	OBP12	−0.69	3.86	0.001
	OBP11	0.21	0.86	0.404
	EAG values			
	Mp 0.1 mg/mL	−0.76	4.72	<0.001
	Mp 1.0 mg/mL	−0.70	3.88	0.001
	Mp 10.0 mg/mL	−0.83	5.88	<0.001
	Eg 0.1 mg/mL	0.05	0.22	0.829
	Eg 1.0 mg/mL	−0.28	1.17	0.261
	Eg 10.0 mg/mL	−0.11	0.44	0.663

Mp = *Mentha piperita*; Eg = *Eucalyptus globulus*; r_s_ = Spearman’s coefficient correlation.

## Data Availability

Not Applicable.

## Supplementary Materials

The following are available online at https://www.mdpi.com/article/10.3390/insects12100895/s1, Figure S1: Gene expression of other OBPs analyzed in antennae of worker bees of different ages that were inoculated (I-DWV) and non-inoculated (N-DWV) with DWV-A. Figure S2: DWV-A load measured in worker honey bees (without antennas) of different ages that were inoculated (I-DWV) and non-inoculated (N-DWV).
