# Supplementary material for: Variant A of the Deformed Wings Virus Alters the Olfactory Sensitivity and the Expression of Odorant Binding Proteins on Antennas of Apis mellifera
Source: Insects. 2021 Oct 1;12(10):895. doi: 10.3390/insects12100895 (PMC8541218; doi:10.3390/insects12100895)
Supplement: Supplementary file 1 [file insects-12-00895-s001.zip › insects-1320483-supplementary.pdf]

insects

MDPI

Supplementary Material: Variant A of the Deformed Wings Virus Alters the Olfactory Sensitivity and the Expression of Odorant Binding Proteins on Antennas of *Apis mellifera*

Diego Silva [1], Ricardo Ceballos [2], Nolberto Arismendi [3], Anne Dalmon [4] and Marisol Vargas [1,*]

[1] Laboratorios de Virología y Patologías en Abejas, Facultad de Agronomía, Universidad de Concepción, Av. Vicente Méndez 595, Chillán, postcode 3780000, Chile; diegosilva@udec.cl
[2] Laboratorio de Ecología Química, Instituto de Investigaciones Agropecuarias, INIA Quilamapu, Av. Vicente Méndez 515, Chillán, postcode 3780000, Chile; rceballos@inia.cl
[3] Centro de Investigación Austral Biotech, Facultad de Ciencias, Universidad Santo Tomás, Av. Picarte 1130–1160, Valdivia, postcode 5090000, Chile; narismendi@santotomas.cl
[4] Unité de Recherche Abeilles et Environnement,, INRAE, F-84000 Avignon, France; anne.dalmon@inrae.fr
* Correspondence: marisolvargas@udec.cl

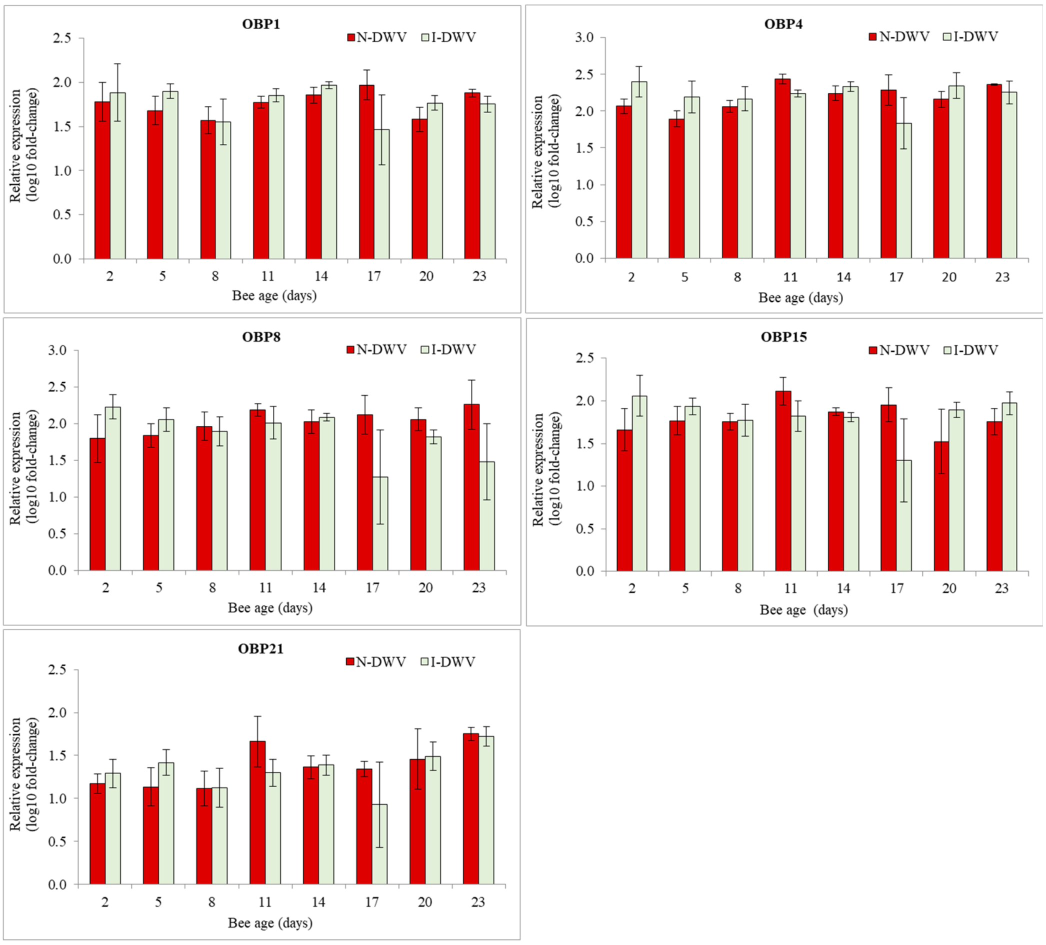

Figure S1. Gene expression of other OBPs analyzed in antennae of worker bees of different ages that were inoculated (I-DWV) and non-inoculated (N-DWV) with DWV-A. Bars in each graph indicate standard error.

insects

MDPI

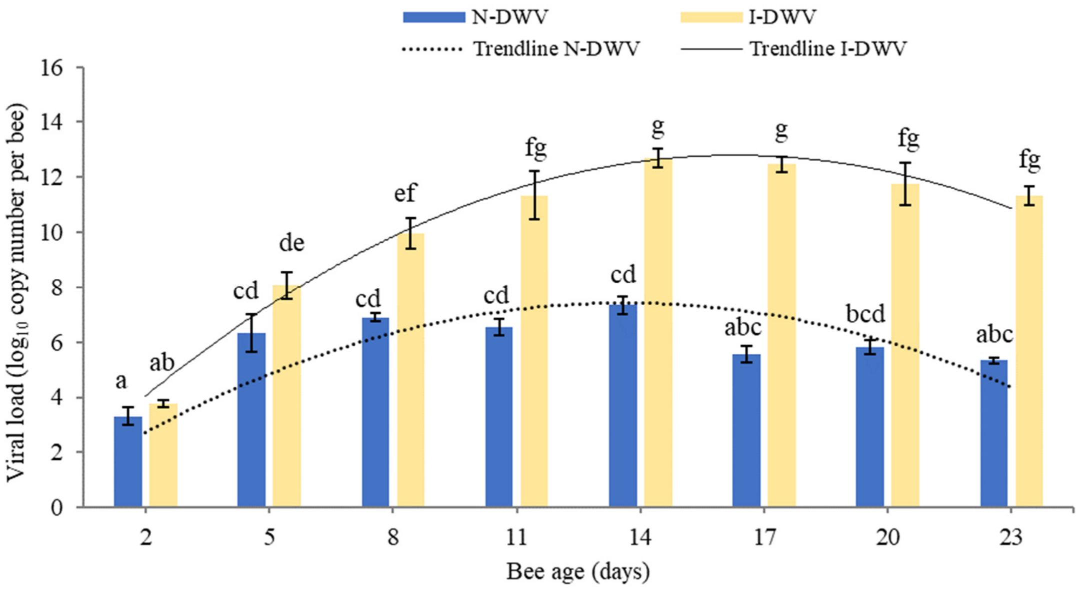

Figure S2. DWV-A load measured in worker honey bees (without antennas) of different ages that were inoculated (I-DWV) and non-inoculated (N-DWV). Difference between treatments was determinate by Factorial ANOVA ($F = 13.22$; $df = 7, 32$; $p < 0.001$). Means (±SE) with different letters indicate significant differences according to the Tukey HSD test ($p < 0.05$). Values of DWV-A load in 2 day-old bees indicate the basal level of viral infection before the inoculation.
